# Genetic and epigenetic heterogeneity of epithelial ovarian cancer and the clinical implications for molecular targeted therapy

**DOI:** 10.1111/jcmm.12771

**Published:** 2016-01-22

**Authors:** Huimin Bai, Dongyan Cao, Jiaxin Yang, Menghui Li, Zhenyu Zhang, Keng Shen

**Affiliations:** ^1^ Department of Obstetrics and Gynecology Beijing Chao‐Yang Hospital Capital Medical University Beijing China; ^2^ Department of Obstetrics and Gynecology Peking Union Medical College Hospital Chinese Academy of Medical Sciences & Peking Union Medical College Beijing China

**Keywords:** epithelial ovarian cancer, EOC, tumoural heterogeneity, TH, genetic and epigenetic alterations, molecular targeted treatment

## Abstract

Epithelial ovarian cancer (EOC) is the most lethal gynaecological malignancy, and tumoural heterogeneity (TH) has been blamed for treatment failure. The genomic and epigenomic atlas of EOC varies significantly with tumour histotype, grade, stage, sensitivity to chemotherapy and prognosis. Rapidly accumulating knowledge about the genetic and epigenetic events that control TH in EOC has facilitated the development of molecular‐targeted therapy. Poly (ADP‐ribose) polymerase (PARP) inhibitors, designed to target homologous recombination, are poised to change how breast cancer susceptibility gene (BRCA)‐related ovarian cancer is treated. Epigenetic treatment regimens being tested in clinical or preclinical studies could provide promising novel treatment approaches and hope for improving patient survival.

## Introduction

Human epithelial ovarian cancer (EOC) is the most common cause of death from gynaecological malignancy 1. The standard treatment for EOC involves cytoreductive surgery followed by chemotherapy consisting of platinum and taxol. For high‐grade serous ovarian cancer (HGSOC), the most prevalent and aggressive form of EOC, relapse is nearly the norm due because of the development of resistance, although approximately 80% of patients initially respond to treatment 2. Tumoural heterogeneity (TH) has been blamed for this treatment failure 3. Gerlinger and Swanton 4 reported that genetic TH fosters the development of cancer drug resistance through Darwinian evolution, which points to a promising therapeutic target for preventing the evolution of more aggressive or resistant clones.

With the advent of next‐generation sequencing in recent years, EOC has been found to consist of a complex set of diseases. Diverse genetic or epigenetic alterations that are of fundamental importance in tumorigenesis and progression have been identified in heterogeneous subsets of patients 5. For example, breast cancer susceptibility gene (BRCA) mutations are most commonly associated with HGSOC 6. Determining the molecular events that control this tumour trait might advance our understanding of tumorigenesis and facilitate individualized treatment strategies for this lethal disease.

## Molecular portraits underlying TH of EOC

Underlying the hallmarks of cancers is genome instability, which can generate genetic diversity 7. Genetic alterations can potentially upset the balance between proto‐oncogenes and tumour suppressor genes, leading to tumorigenesis. The existence of extensive cytogenetic, genetic and epigenetic variations has been reported in EOC cell populations.

Numerical or structural chromosomal abnormalities are frequently observed in almost all human tumours 7. Rearrangement of 19q has been identified in 61.6% of patients with ovarian cancer; such rearrangements have been significantly correlated with high‐grade tumours, predicting shorter disease‐free survival and worse overall survival (OS) 8 (Table 1). Underrepresentation of 11p and 13q and overrepresentation of 8q and 7p have been significantly correlated with undifferentiated ovarian carcinomas 9. Underrepresentation of 12p and overrepresentation of 18p are frequently identified in well‐ and moderately differentiated ovarian tumours. Patients showing loss of D6S1581 are more likely to be resistant to platinum‐based chemotherapy 10. Gains of 14q32.33 have been associated with platinum resistance and reduced progression‐free survival (PFS) and OS for patients with EOC 11. Tumours exhibiting gain of 2p22‐p25, 19p12‐q13.1, and 20q12‐q13 and loss of 5q14‐q22 present a high risk of recurrence. The OS of patients is inversely correlated with the number of chromosomal alterations found in their tumours 12. Gains at 5p are adversely associated with tumour recurrence 13, and gains at 1p and losses at 5q are associated with a significant decrease in recurrence. Loss at 6q24.2‐26 is independently associated with a cluster of patients with HGSOC showing longer survival 14.

**Table 1:** Cytogenetic and genetic tumour heterogeneity in EOC

Molecular events	Heterogeneous clinicopathological characteristics				
	Histology	Grade	Response to CT	Relapse risk	Survival
Chromosomal abnormalities					
Rearrangement of 19q 8	HGSOC			High	Adverse
Underrepresentation of 11p and 13q; overrepresentation of 8q and 7p 9		High			
12p underrepresentation and 18p overrepresentation 9		Low			
Loss of D6S1581 10			Resistant		
Gains of 14q32.33 11			Resistant	High	Adverse
Gains of 2p22p25, 19p12q13.1 and 20q12q13 and loss of 5q14q22 12				High	Adverse
Gain in 5p 13				High	
Gain in 1p and loss in 5q 13				Low	
Loss at 6q24.2‐26 14					Favourable
Gene copy number variation					
Gains of FGF3/4 and CCNE1 18	Serous				
KRAS amplification 17	HGSOC, rare in mucinous tumour				
Gain of JUNB, KRAS2, MYCN, ESR and CCND2 18; TPM3 amplification 17	Endometrioid				
ERBB2 amplification 17	Mucinous				
Amplification of FGFR1 and MDM2; gain of PIK3CA 18		Borderline			
PIK3CA amplification 20			Sensitive		
CCNE1amplification 20, 21; Akt/AKT1 overexpression 23			Resistant		
AKT2 amplification		High 22	Resistant 23		Adverse 22
Amplification of KLK6 24, EGFR 25, LMX1B 26, BMP8B, and ATP13A4 27				High	Adverse
GAB2 amplification 27			Sensitive	Low	Favourable
Somatic gene mutation					
TP53 15, 20, 28; FAT3, CSND3, NF1, CDK12, RB1, and GABRA6 15	HGSOC				
BRCA1/2	HGSOC 6		Sensitive 39, 40		Favourable 39, 40
Reversions of germline BRCA1 or BRCA2 mutations or loss of BRCA1 promoter methylation 20, 41, 42			Resistant		Adverse
BRAF	Not mucinous 34; Serous Borderline tumours 33				
KRAS 17, 34	Mucinous	High			
PTEN loss 35; PIK3CA mutation with gain of function 36	Endometrioid and clear cell carcinoma				
LRP1B deletion 38	HGSOC		Resistant		

CT: chemotherapy; HGSOC: high‐grade serous ovarion cancer; EOC: epithelial ovarian cancer.

Gene copy number variations generally result in the abnormal expression of genes that are located within rearranged chromosomal regions. Nonrandom gains and deletions of DNA copy numbers and imbalances of alleles are frequently identified in ovarian tumours 15, 16. Somatic copy number amplification is highly prevalent in high‐grade ovarian cancer, whereas somatic mutational activation of oncogenes is a rare event, suggesting that the former is a common mechanism 17 of oncogene activation in this tumour type 15. In addition, variations in gene copy number are specific to tumour histotypes, among which serous is the most prevalent, followed by endometrioid, clear cell and mucinous 17. Mayr *et al*. 18 demonstrated that gains of FGF3/4 and CCNE1 occur in all serous carcinomas. Endometrioid carcinomas most frequently show gains of JUNB, KRAS2, MYCN, ESR and CCND2. Among serous borderline tumours, 80% exhibit amplification of FGFR1 and MDM2, and 75% show gains of PIK3CA (Table 1). By applying an *in silico* hypothesis‐driven approach to multiple datasets, Huang *et al*. 17 found 76 cancer genes to be significantly altered in EOC, several of which may be potential copy number drivers, such as ERBB2 in mucinous tumours and TPM3 in endometrioid histotypes. In addition, KRAS was observed to be significantly amplified in serous tumours, although mutations are rare in such high‐grade tumours. Copy number variations can also predict a patient's prognosis and response to treatment. Patients showing PIK3CA amplification generally respond well to treatment 19. In contrast, amplification of 19q12 involving CCNE1 is the dominant structural variant associated with primary treatment failure of patients with HGSOC 20, 21. Amplification of AKT2 is frequently identified in undifferentiated tumours and predicts a poor prognosis^22^. Ovarian cancer cells that either constitutively overexpress active Akt/AKT1 or exhibit AKT2 gene amplification are highly resistant to paclitaxel compared with cells with low AKT levels 23. Overexpression of KLK6 24, EGFR 25, LMX1B 26, BMP8B and ATP13A4 27, because of gene amplification or high copy number gains, is associated with worse PFS and OS in patients with ovarian cancer. In contrast, an increased copy number of GAB2 is associated with improved PFS and OS and correlates with enhanced sensitivity to the dual PI3K/mTOR inhibitor PF‐04691502 *in vitro* 27.

TP53 mutations are almost invariably present in HGSOC 15, 18, 20 (Table 1). The early loss of P53 function observed in sporadic cancers could create a permissive environment for the loss of BRCA1 or BRCA2 function (or other phenotypes of DNA repair deficiency), which would otherwise lead to apoptosis because of checkpoint activation 29. Inactivation of BRCA1 and/or BRCA 2 is detected in 67% of patients with HGSOC, which is markedly higher than in the other histotypes of EOC 6. However, only 7–9% of sporadic ovarian cancers exhibit BRCA1 30 mutations leading to inactivation of BRCA1, while 4% exhibit BRCA2 mutations 31. HGSOC tumours only form in animal models when all three of the BRCA, TP53 and PTEN genes are altered, which suggests a synergistic role of these genes in tumorigenesis 32. Mutation in other genes, including FAT3, CSND3, NF1, CDK12, RB1 and GABRA6, are also frequently identified in HGSOC tumours 15. Mutations in BRAF are restricted to serous borderline tumours, indicating that the majority of serous borderline tumours do not progress to serous carcinomas 33. Activating KRAS mutations are more common in mucinous tumours than in all other histological types 17, 34, while no mucinous tumours have been found to harbour a BRAF mutation 34. Loss or dysfunction of mismatch repair of gain‐of‐function PTEN 35 and PIK3CA 36 mutations is common in endometrioid and clear cell carcinoma, but not in serous or mucinous ovarian cancer 37. Deletion of LRP1B in HGSOC is associated with acquired resistance to liposomal doxorubicin 38. In addition to their histological implications, tumours with BRCA mutations are more likely to be platinum‐sensitive and associated with longer PFS and OS 39, 40. Reversion of germline BRCA1 or BRCA2 mutations in individual patients or loss of BRCA1 promoter methylation predicts resistance to platinum 20 and may also predict resistance to PARP ((poly (ADP‐ribose) polymerase) inhibitors 41, 42.

Epigenetics is defined as heritable changes in gene expression that do not alter the DNA sequence itself. The mechanisms responsible for such changes include DNA methylation, histone modification, and microRNAs, which are related to post‐transcriptional gene regulation. Epigenetic alterations are increasingly being implicated in the development and progression of ovarian cancer, and the gradual accumulation of epigenetic alterations has been associated with an advancing grade and stage of disease 43 (Table 2).

**Table 2:** Epigenetic tumour heterogeneity in EOC

Molecular events	Heterogeneous clinicopathological characteristics					
	Histology	Grade	Stage	Response to CT	Relapse risk	Survival
Hypomethylation						
Satellite DNA hypomethylation 47		High	Advanced			Adverse
Re‐expression of MCJ, SNCG, and BORIS 45; overexpression of CLDN4, MAL, BORIS, and TUBB3 44				Resistant		
LINE‐1 54 and CT45 55		High	Advanced		High	Adverse
HOXA10 promoter hypomethylation	CCC 52, 53, rare in serous tumour 53					Adverse 53
Hypermethylation or methylation						
MLH1Hypermethylation 57		High		Resistant		
hMLH1 promoter methylation				Resistant 58, 59, 60	High 59	Adverse 59
hMSH2 57	Endometrioid	High				
DLEC1 methylation 60	HGSOC		Advanced		High	
FBXO32 promoter hypermethylation or methylation 62			Advanced	Resistant	High	
Promoter hypermethylation of ARMCX2, COL1A1, MDK, and MEST 60				Resistant		
BRCA1 promoter hypermethylation	Serous 67, 68			Resistant 20		Adverse 68
Histone modification						
H3‐K27 m3 loss		High 78	Advanced 78	Resistant 79, 80		Adverse 78
Proportion of SIRT1 expression 79	Serous					
SIRT1 overexpression 81	Serous		Early			Faverable
MiRNAs						
Up‐regulation of miR‐205 85		High	Advanced			
Up‐regulation of miR‐200a 86		High	Advanced		High	
Down‐regulation of miR‐101 87		High	Advanced	Resistant		
Reduced expression of miR‐34b*/c 88, hsa‐miR‐200a, hsa‐miR‐34a, and hsa‐miR‐449b 89			Advanced			
Up‐regulation of Hsa‐miR‐378 89				Sensitive		
Reduced expression of miR‐30c, miR‐130a, miR‐335 91, and miRNA‐149 94; overexpression of MiR‐214 92 and MiR‐197 94				Resistant		
Overexpression of miR‐200c 86					High	
Reduced expression of let‐7i 90				Resistant	High	
Overexpression of miR‐200, miR‐141, miR‐18a, miR‐93, and miR‐429 95						Favourable
Overexpression of hsa‐miR‐27a 89, let‐7b, and miR‐199a 95						Adverse

Methylation, which consists primarily of demethylation of oncogenes and hypermethylation of tumour suppressing genes, is frequently identified in ovarian cancer 44, 45. Gene hypermethylation and satellite and global DNA hypomethylation in ovarian tumours are both independently associated with the degree of malignancy 46. Satellite DNA hypomethylation is significantly more prevalent in advanced‐stage and high‐grade ovarian cancers and is an independent marker of poor prognosis 47. In addition to repetitive elements and DNA satellites, hypomethylation of promoter CpG islands and gene overexpression have been reported in ovarian cancer. CpG islands are DNA sequences containing CpG sites at an atypically high frequency 48 and are usually, but not exclusively, associated with gene promoters 49. Demethylation of CpG islands in gene promoters generally allows active gene transcription to occur 50. As a result of hypomethylation, re‐expression of MCJ, SNCG, and BORIS and overexpression of CLDN4, MAL, BORIS 45 and TUBB3 44 have been associated with chemoresistance in patients with EOC. As a result of promoter hypomethylation 51, HOXA10 is overexpressed in ovarian clear cell adenocarcinomas, but not in ovarian serous adenocarcinomas, normal ovarian epithelia or endometrial cysts 53. In addition, this overexpression in ovarian clear cell adenocarcinomas 52, 53 is associated with poor survival 53 . DNA hypomethylation‐mediated activation of the LINE‐1 54 and CT45 55 genes is correlated with high‐grade and advanced‐stage EOC and associated with poorer PFS and OS.

Aberrant methylation of CpG islands in ovarian tumours is associated with silencing of genes involved in the control of the cell cycle, apoptosis and drug sensitivity as well as tumour suppressor genes 56. Hypermethylation of the MLH1 gene, accompanied by loss of gene expression, and methylation of hMSH2 are correlated with a higher histological grade and lymph node metastasis of EOC 57. In addition, methylation of the hMLH1 promoter has been identified in 56% of EOC patients with acquired resistance to platinum‐based chemotherapy 58, 59, 60, predicting a high risk of relapse and poor OS 59. The methylation rate of hMSH2 is significantly higher in endometrioid adenocarcinoma tissues compared with other histological types of the disease 57. Epigenetic silencing of ARMCX2, COL1A1, MDK and MEST due to promoter hypermethylation at CpG sites has also been linked to the development of platinum‐based resistance in ovarian cancer 60. Methylation of DLEC1 is associated with recurrence of HGSOC, independent of tumour stage and suboptimal surgical debulking 61. Chou *et al*. 62 reported that hypermethylation of the FBXO32 promoter is more commonly observed in advanced‐stage ovarian tumours, and patients showing FBXO32 methylation exhibit significantly shorter PFS. Re‐expression of FBXO32 was demonstrated to markedly reduce proliferation, increase apoptosis, and restore sensitivity to cisplatin in a platinum‐resistant ovarian cancer cell line both *in vitro* and *in vivo*.

BRCA1 and BRCA2 germline mutations are present in the majority of patients with hereditary ovarian carcinoma 63, in contrast to the frequency of these mutations detected in unselected patients, which is only 15.3% 64. The majority of ovarian cancers arise independently of mutations in the BRCA1/2 genes 65. BRCA1/2 alterations of all kinds, including mutations, have been reported in up to 82% of ovarian tumours 31. The term ‘BRCAness’ has been used to describe the phenotypic traits that some sporadic ovarian tumours share with tumours found in BRCA1/2 germline mutation carriers and reflects similar causative molecular abnormalities 66. BRCAness appears to be the result of different epigenetic processes. Recent data suggest that hypermethylation of the BRCA1 promoter occurs in 10‐15% of sporadic cases and is associated with the serous histotype 67, 68. BRCA2 can also be down‐regulated through silencing of its upstream regulator, FANCF, by promoter methylation 69, 70. Although patients with BRCA1/2 mutations and low protein/mRNA expression of BRCA1 tend to show a favourable response to treatment^20^ and a better outcome 40, BRCA1 promoter methylation is significantly correlated with resistance to treatment 20 and a poorer prognosis 68 in patients with EOC. Thus, methylation is not functionally equivalent to a germline mutation in mediating chemotherapy sensitivity. While methylation of BRCA1 is common in sporadic ovarian cancer, it has not been reported in the hereditary form of the disease or in samples from women with germline BRCA1 mutations 71. BRCA2 does not present a similar methylation profile in ovarian cancer 72.

DNA‐associated histone proteins are subject to extensive modifications that mediate the assembly of transcriptionally permissive or repressive (*i.e*., open or closed) chromatin. Chromatin modifiers regulate the expression of different sets of genes involved in tumorigenesis 73. DNA methylation and histone deacetylation often coordinately inhibit gene transcription 74. However, histone modification is an independent mechanism of epigenetic gene regulation under some conditions 75, 76. H3K27m3 is a transcription‐suppressive histone mark found in chromatin in association with EZH2, a component of the Polycomb (PcG) complex 77. In ovarian cancer, decreased expression of H3K27me3 is significantly associated with high‐grade and advanced‐stage tumours, but not with the histological type 78, predicting resistance to chemotherapy 79 and a poor clinical outcome in ovarian cancer and other malignancies 78. Removal of H3K27 methylation was shown to lead to re‐expression of the RASSF1 tumour suppressor and resensitize drug‐resistant ovarian cancer cells to cisplatin; this increased platinum access to DNA was likely due to relaxation of condensed chromatin 80. Sirtuin1 (SIRT1) is a nicotinamide adenine dinucleotide‐dependent deacetylase and a class III histone deacetyltransferase. The proportion of SIRT1 expression is significantly higher in serous carcinoma compared with mucinous tumours. SIRT1 overexpression is more common in early‐stage serous carcinomas and is correlated with longer OS compared with late‐stage disease 81. SIRT1 also facilitates the acquisition of drug resistance through its influence on the tumour microenvironment, function in DNA repair and promotion of cancer stem cell survival 82. Thus, SIRT1 is being considered as a possible target for overcoming drug resistance in many malignancies.

Having been implicated in the initiation and progression of human cancers, microRNAs regulate processes such as cell growth, differentiation and apoptosis 83. A variety of miRNAs are associated with tumour subtype, stage, grade, therapy resistance and prognosis in ovarian cancer 84 (Table 2). Up‐regulation of miR‐205 85 and miR‐200a 86 and down‐regulation of miR‐101^87^ are significantly associated with a high pathological grade and advanced stage of EOC in patients. In addition, patients with lymph node metastasis show significant elevation of miR‐200c 86. Reduced expression of miR‐34b*/c 88, hsa‐miR‐200a, hsa‐miR‐34a and hsa‐miR‐449b 89 is frequently identified in advanced‐stage tumours. Hsa‐miR‐378 89 and let‐7i 90 are up‐regulated in patients who are sensitive to platinum; in contrast, miR‐101, 87 miR‐30c, miR‐130a and miR‐335 91 are down‐regulated in several resistant ovarian cancer cell lines, suggesting direct involvement in the development of chemoresistance. MiR‐214 induces cell survival and cisplatin resistance through targeting the 3′‐UTR of the PTEN gene, which leads to reduced expression of PTEN and activation of the Akt pathway 92. Down‐regulation of miRNA‐149 decreases the sensitivity of ovarian cancer cells to paclitaxel treatment by increasing MyD88 expression 93. MiR‐197 is significantly increased in Taxol‐resistant ovarian cancer cells 94. In addition, decreased expression of let‐7i 90 and overexpression of miR‐200a and miR‐200c 86 are associated with shorter PFS, suggesting their potential for predicting relapse. Overexpression of miR‐200, miR‐141, miR‐18a, miR‐93 and miR‐429 95 is associated with improved OS, whereas high levels of hsa‐miR‐27a, 89 let‐7b and miR‐199a 95 are potentially correlated with a poor prognosis in patients with EOC.

## Molecular targeted treatment

The rapid development of genetics and epigenetics has facilitated the study of the molecular mechanisms of TH in EOC. This knowledge has led to the introduction of novel treatments that are rationally designed to target specific molecular factors implicated in tumour growth (Table 3).

**Table 3:** Molecular‐targeted treatments for EOC

Drug	Condition	Treatment regimen	Trial phase
Targeting homologous recombination (PARP inhibitors)			
Olaparib 101, 102	BRCA‐associated ovarian cancer in both newly diagnosed and platinum‐sensitive recurrent settings	Combined with post‐platinum based CT	Phase III
Veliparib 101	Recurrent HGSC (both germline BRCA and sporadic allowed	Combined with Temozolomide	Phase II
Niraparib 101	Recurrent platinum‐sensitive ovarian cancer	Combined with post‐platinum based CT	Phase III
Rucaparib 101	Recurrent platinum‐sensitive ovarian cancer	Combined with post‐platinum based CT	Phase III
BMN673 101	Advanced or recurrent EOC	Single agent	Phase I
Targeting the PI3K/AKT/mTOR pathway			
Perifosine 110	Recurrent EOC	Combined with docetaxel	Phase II
Temsirolimus 111	Primary, persistent or recurrent EOC	Single agent	Phase II
Targeting aberrant DNA methylation			
Cytarabine 114	CT‐resistant EOC	Single agent	Preclinical
Zebularine 115	CT‐resistant EOC	Combined with cisplatin	Preclinical
Azacitidine 116	Platinum‐resistant	Combined with platinum	Phase Ib‐IIa
Decitabine 117	Recurrent or platinum‐resistant EOC	Combined with platinum	Phase I
Targeting histone modifications			
Vorinostat 121	Persistent or recurrent EOC	Single agent	Phase II
Romidepsin 122	Related data not available		Phase II
Valproate 123, 124	Primary or resistant EOC	Single agent or combined with platinum	Preclinical
PXD101 125	CT‐resistant EOC	Combined with platinum	Preclinical
Targeting miRNA dysregulation			
MiR‐124 129	Advanced EOC	Single agent	Preclinical

Dysfunction of BRCA1 and BRCA2 is associated with ovarian cancer tumorigenesis, due to an inability to repair DNA double‐strand breaks (DSBs) 96. The PARPs are a family of enzymes involved in base excision repair, a key pathway in the repair of DNA single‐strand breaks (SSBs). PARP inhibition leads to the persistence of spontaneously occurring SSBs and subsequent formation of DSBs, as the SSBs stall and collapse replication forks. These DSBs cannot be repaired by the defective HR pathway in BRCA‐mutated cells, resulting in cell death.

PARP inhibitors induce synthetic lethality in BRCA‐deficient tissues. BRCA1/2‐deficient cancers are now recognized as the target of a class of drugs known as PARP inhibitors. Deficiency of either PARP or BRCA alone has no impact, but deficiency in both leads to a lethal effect 97, 98. Clinical investigation of the use of PARP inhibitors for the treatment of EOC evolved rapidly from the observations of single‐agent activity conducted *in vitro* in BRCA‐deficient cancer cells in 2005 to the initiation of multiple phase 3 studies in 2013. Ledermann *et al*. 99 retrospectively analysed the data from a randomized, double‐blind, phase 2 study 100 and showed that patients with recurrent, platinum‐sensitive serous ovarian cancer with a BRCA mutation exhibit the highest likelihood of benefiting from olaparib, the first human PARP inhibitor. Two phase III studies have been carried out to test olaparib *versus* placebo as maintenance therapy for both newly diagnosed and platinum‐sensitive recurrent BRCA‐associated ovarian cancer 101. In December 2014, olaparib was approved for the treatment of patients with germline BRCA1/2‐associated advanced ovarian cancer who have received three or more lines of chemotherapy. This approval represents the first ‘personalized’ therapy for ovarian cancer 102. Other PARP inhibitors that have been tested or are currently being tested in clinical trials for ovarian cancer include veliparib, niraparib, rucaparib and BMN673 101. In addition to ovarian cancer, PARP inhibitors have shown encouraging in for other BRCA1/2 mutation‐related cancers, such as breast cancer 103, endometrial cancer 104, prostate cancer 105 and pancreatic cancer 106. Future and ongoing trials will identify the most effective role of these agents for use in human cancer treatment.

The signalling cascade involving PI3K, AKT and mTOR plays a key role in mediating cell proliferation and survival and is one of the pathways that is frequently affected in human cancer 107. Various genetic alterations that activate PI3K/AKT/mTOR signalling have been identified in ovarian cancer 108. In a previous study, we demonstrated that PI3K/AKT/mTOR pathway activation is associated with significantly higher migratory and invasive capacities in subpopulations of human ovarian cancer cell lines 109. Thus, this pathway is regarded as an attractive candidate for therapeutic interventions against EOC, and inhibitors targeting different components of the pathway are in various stages of clinical development. Thus far, results have been published only for a phase I trial of an AKT inhibitor, perifosine 110, and a phase II trial of an mTORC1 inhibitor, temsirolimus 111. Perifosine plus docetaxel appears to be effective in patients with mutational activation of the PI3K/AKT pathway 110. A phase II clinical trial is currently being conducted to investigate the efficacy of perifosine as well as the association between PIK3CA status and the response to treatment in patients with recurrent gynaecological malignancies, including ovarian cancer. In a GOG phase II trial, 111 temsirolimus monotherapy showed modest activity in persistent or recurrent EOC and primary peritoneal cancer, and PFS was just below that required to warrant the inclusion of unselected patients in phase III studies. Based on these results, a phase II trial is currently being conducted specifically targeting ovarian clear cell carcinoma, which often exhibits PI3K/AKT/mTOR activation 108. This trial is aimed at examining the use of temsirolimus in combination with carboplatin and paclitaxel, followed by temsirolimus consolidation, as a first‐line therapy for patients with ovarian cancer, and its results appear promising.

Because genetic alterations are almost impossible to reverse, the potential reversibility of epigenetic mechanisms makes them more attractive candidates for the prevention and treatment of ovarian carcinoma 112. There are two types of DNA methylation inhibitors (DNMTIs): nucleoside and non‐nucleoside analogues 44. Nucleoside analogues, such as cytarabine and decitabine, can inhibit methylation when they are integrated into DNA and block the release of DNA methyltransferases by forming a covalent complex with these enzymes 113. Cytarabine has been reported to induce re‐expression of hMLH1 and reverse drug resistance in human tumour xenografts through demethylation of the hMLH1 promoter 114. Zebularine can also induce demethylation of hMLH1 and RASSF1A and resensitize drug‐resistant cell lines to cisplatin 115. The ability of azacitidine and decitabine to reverse platinum resistance in ovarian cancer patients has been preliminarily confirmed in two clinical trials 116, 117.

Inhibitors of histone deacetylation (HDACIs) represent another promising new class of anticancer agents. Among the currently available HDACIs, four have been tested in ovarian cancer, including vorinostat, romidepsin, valproate and PXD101. Vorinostat and romidepsin have both been approved by the FDA for the treatment of cutaneous T‐cell lymphoma. Both agents, in combination with cytotoxic agents, have shown significant activity in inhibiting ovarian cancer cell growth in preclinical studies 118, 119, 120. However, in a phase II study, vorinostat displayed minimal activity as a single agent for treating persistent or recurrent epithelial ovarian or primary peritoneal carcinoma, despite its acceptable tolerability 121. A phase II trial examining the use of romidepsin for the treatment of ovarian cancer is ongoing 122. Valproate exhibits direct HDACI activity, although the associated mechanisms of action remain unclear. Valproate is effective in sensitizing ovarian cancer cells to cisplatin and resensitizing cisplatin‐resistant cells, both alone and in combination with other drugs 123, 124. PXD101 can increase the acetylation of A‐tubulin induced by docetaxel and the phosphorylation of H2AX induced by carboplatin. In addition, this drug can effectively reverse drug tolerance in both *in vitro* and *in vivo* models of ovarian cancer 125.

DNA methylation and histone modifications are intimately linked 74. Hence, combining two classes of epigenetic drugs, DNMTIs and HDACIs, with conventional therapies may be a more effective approach in the clinic 126.

The dysregulation of miRNA expression in tumours makes miRNAs another potential therapeutic target, necessitating the specific identification of genes that are targets of miRNA regulation. The overexpression of miRNAs that act as oncogenes can be targeted for down‐regulation through the use of anti‐miRNA oligonucleotides, miRNA masking, miRNA sponges or small molecule inhibitors. In contrast, restoring the activity of tumour suppressor miRNAs can inhibit proliferation and induce apoptosis of tumour cells, and miRNA mimics are applicable under these conditions 127. Several clinical trials have been initiated to test the efficacy of miRNA‐based therapeutics for the treatment of leukaemia, prostate cancer, and skin cancer 128 . As for ovarian cancer, this therapeutic approach is still at a preclinical stage to the best of our knowledge. Having identified miR‐124 as a potential tumour suppressor that can functionally target the p27/myc/phospho‐Rb protein signature, Seviour *et al*. 129 demonstrated that nanoparticle‐mediated delivery of miR‐124 can reduce tumour growth and sensitize cells to etoposide in a xenograft model. These findings present an exciting opportunity for the potential therapeutic use of miR‐124 in combination with chemotherapy in patients with late‐stage EOC.

## Conclusions

Epithelial ovarian cancer is a heterogeneous disease. As discussed above, the genomic and epigenomic atlas of EOC varies significantly with tumour histotypes, grades and stages as well as with a patient's prognosis and sensitivity to chemotherapy. The rapidly increasing knowledge about the genetic and epigenetic events that control TH in EOC is facilitating the development of molecular targeted therapy. PARP inhibitors, which are designed to target HR, are poised to change how BRCA‐related ovarian cancer is treated, representing the first ‘personalized’ therapy for ovarian cancer. Epigenetic treatment regimens being tested in preclinical or clinical studies are giving rise to optimism regarding the improvement of patient survival and may also provide promising novel treatment approaches.

## Disclosure

The authors have no conflict of interest to declare.

## Author contribution

Huimin Bai, Dongyan Cao, Keng Shen and Zhenyu Zhang: Conception and design of the study, assembly, analysis and interpretation of the data, manuscript writing. The other authors: analysis and interpretation of the data.
